# Supplementary material for: A proteomic atlas of insulin signalling reveals tissue‐specific mechanisms of longevity assurance
Source: Mol Syst Biol. 2017 Sep 15;13(9):939. doi: 10.15252/msb.20177663 (PMC5615923; doi:10.15252/msb.20177663)
Supplement: Supplementary file 1 — Expanded View Figures PDF [file MSB-13-939-s001.pdf]

Expanded View Figures

Figure EV1. Tissue-specific proteomic analysis in *Drosophila*.

A Correlation heatmap (Pearson's correlation coefficient) across all tissues (thorax, gut, fat body and brain) and all genotypes (A: w^{Dah}, B: *dfoxo*$^{\Delta 94}$, C: *InsP3-Gal4/UAS-rpr* and D: *InsP3-Gal4/UAS-rpr, dfoxo*$^{\Delta 94}$).

B Tissue-specific PCA plots showing inter-genotype clustering of individual samples.

C Tissue-specific MA plots of log2 fold change and average expression level (LFQ) for all detected (black) and significantly regulated (orange) proteins between *InsP3-Gal4/UAS-rpr* vs. w^{Dah} flies (FDR = 0.1).

D Tissue-specific volcano plots of −log2 *P*-value (adjusted) and log2FC for all detected (black) and significantly regulated (orange) proteins between *InsP3-Gal4/UAS-rpr* vs. w^{Dah} flies (FDR = 0.1).

 © 2017 The Authors

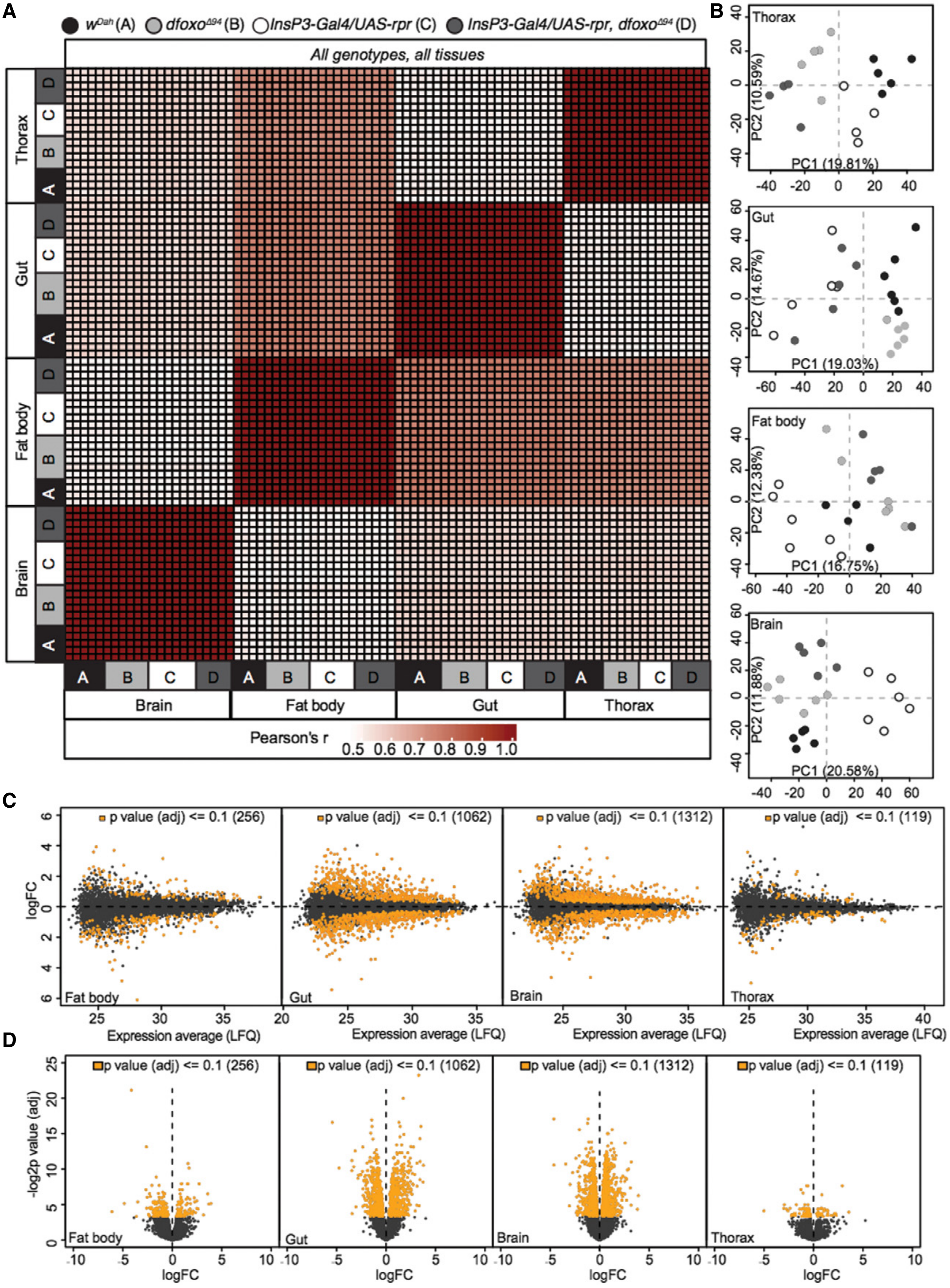
A
wDah (A)
dfoxoΔ94 (B)
InsP3-Gal4/UAS-rpr (C)
InsP3-Gal4/UAS-rpr, dfoxoΔ94 (D)
All genotypes, all tissues
Thorax
Gut
Fat body
Brain
Pearson's r
0.5 0.6 0.7 0.8 0.9 1.0
B
Thorax
PC1 (19.81%)
PC2 (10.59%)
Gut
PC1 (19.03%)
PC2 (14.67%)
Fat body
PC1 (16.75%)
PC2 (12.38%)
Brain
PC1 (20.58%)
PC2 (11.88%)
C
p value (adj) <= 0.1 (256)
p value (adj) <= 0.1 (1062)
p value (adj) <= 0.1 (1312)
p value (adj) <= 0.1 (119)
logFC
Expression average (LFQ)
D
-log2p value (adj)
logFC

Figure EV1.

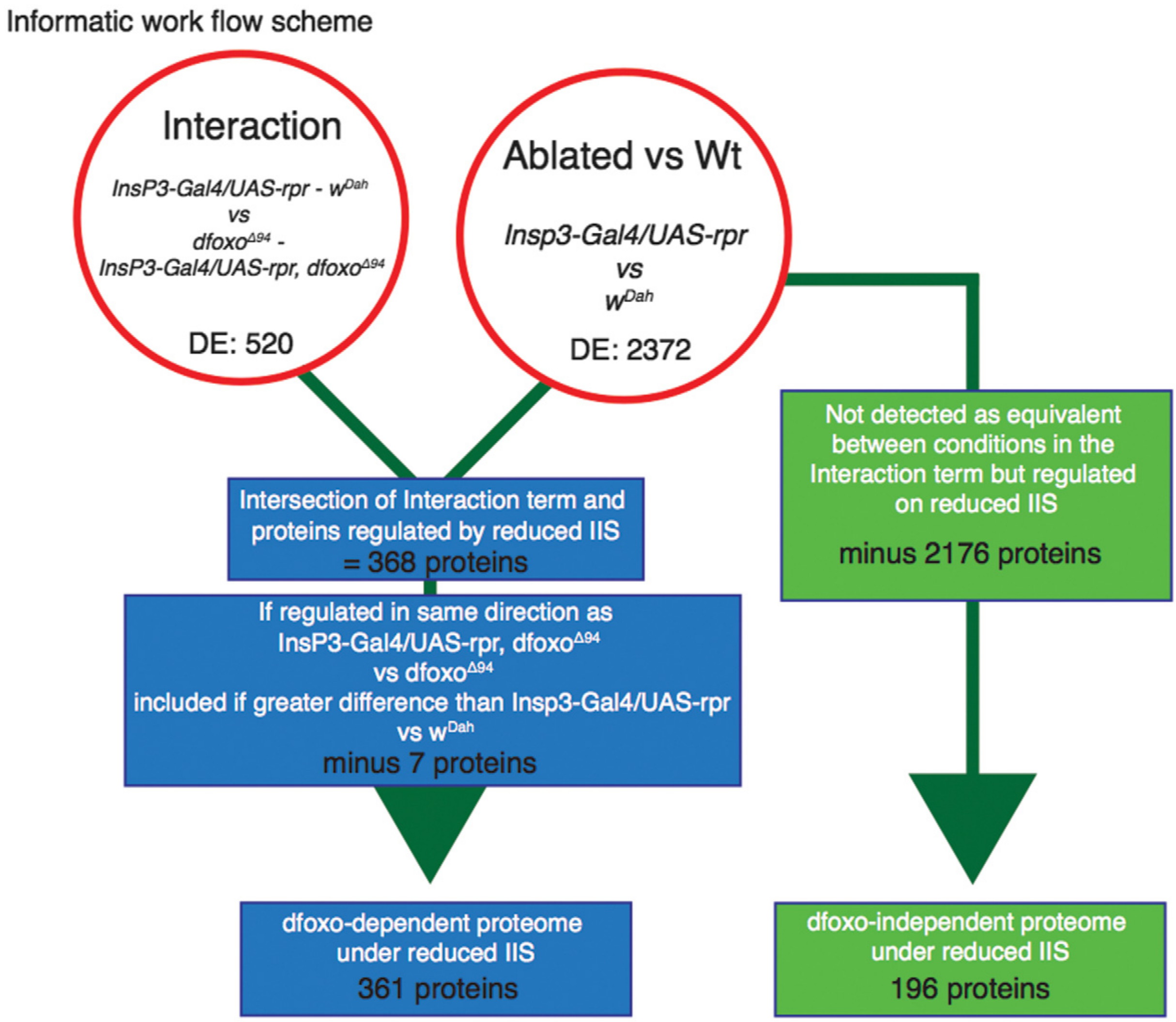

Figure EV2. Bioinformatic scheme for determining *dfoxo*-dependent and -independent protein expression under reduced IIS.

Genotypes defined as ω^{Dah}, *dfoxo*$^{\Delta 94}$ mutant, *InsP3-Gal4/UAS-rpr* and *InsP3-Gal4/UAS-rpr, dfoxo*$^{\Delta 94}$. Reduced IIS-mediated changes in the wild-type background (*InsP3-Gal4/UAS-rpr* vs. ω^{Dah}) and in the *dfoxo*$^{\Delta 94}$ background (*InsP3-Gal4/UAS-rpr, dfoxo*$^{\Delta 94}$ vs. *dfoxo*$^{\Delta 94}$) or their interaction term (*InsP3-Gal4/UAS-rpr* - ω^{Dah}) vs. *InsP3-Gal4/UAS-rpr, dfoxo*$^{\Delta 94}$-*dfoxo*$^{\Delta 94}$. *dfoxo*-dependent proteins were defined as proteins regulated between *InsP3-Gal4/UAS-rpr* and ω^{Dah} and within the interaction term (*InsP3-Gal4/UAS-rpr* - ω^{Dah}vs. *InsP3-Gal4/UAS-rpr, dfoxo*$^{\Delta 94}$-*dfoxo*$^{\Delta 94}$), but also if the expression response was the same in *InsP3-Gal4/UAS-rpr* vs. ω^{Dah} and *InsP3-Gal4/UAS-rpr, dfoxo*$^{\Delta 94}$ vs. *dfoxo*$^{\Delta 94}$ providing the response was greater in *InsP3-Gal4/UAS-rpr* vs. ω^{Dah}. Proteins were identified as *dfoxo*-independent if they were regulated between *InsP3-Gal4/UAS-rpr* and ω^{Dah}, and were not subject to interaction effects (equivalence given a tissue-specific threshold). Also included were proteins with a stronger same directional response in *InsP3-Gal4/UAS-rpr, dfoxo*$^{\Delta 94}$ vs. *dfoxo*$^{\Delta 94}$ than in *InsP3-Gal4/UAS-rpr* vs. ω^{Dah}, regardless of interaction. See Materials and Methods section for full descriptions.

© 2017 The Authors

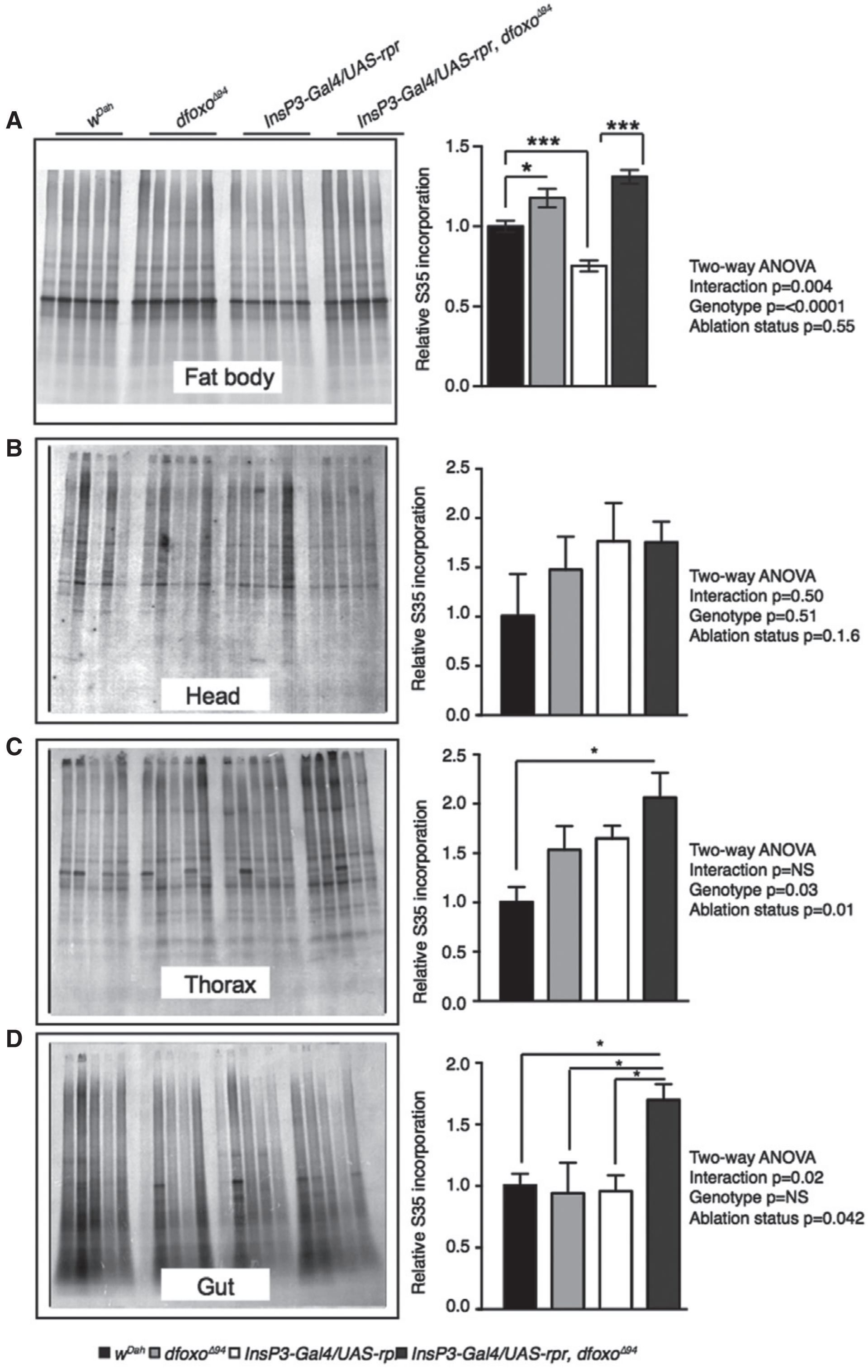

Figure EV3. Translational activity in the fat body under reduced IIS is reduced in a *dfoxo*-dependent manner.

A–D *De novo* protein synthesis as measured by incorporation of ^{35}S into proteins from *ex vivo* (A) fat body, (B) head, (C) thorax and (D) gut tissue. Equal levels of protein were loaded per lane. Representative gel exposures show fat body ^{35}S incorporation from ω^{Dah}, $dfoxo^{\Delta 94}$, *InsP3-Gal4/UAS-rpr* and *InsP3-Gal4/UAS-rpr*, $dfoxo^{\Delta 94}$ flies. Quantification of gel exposures normalized to total protein and corresponding two-way ANOVA, as well as Bonferroni corrected *post hoc* tests are shown alongside each gel exposure. Error bars show SEM, n = 11 for ω^{Dah} and *InsP3-Gal4/UAS-rpr* fat body samples, for all other genotypes and tissues n = 5, *P < 0.05, ***P < 0.001.

© 2017 The Authors

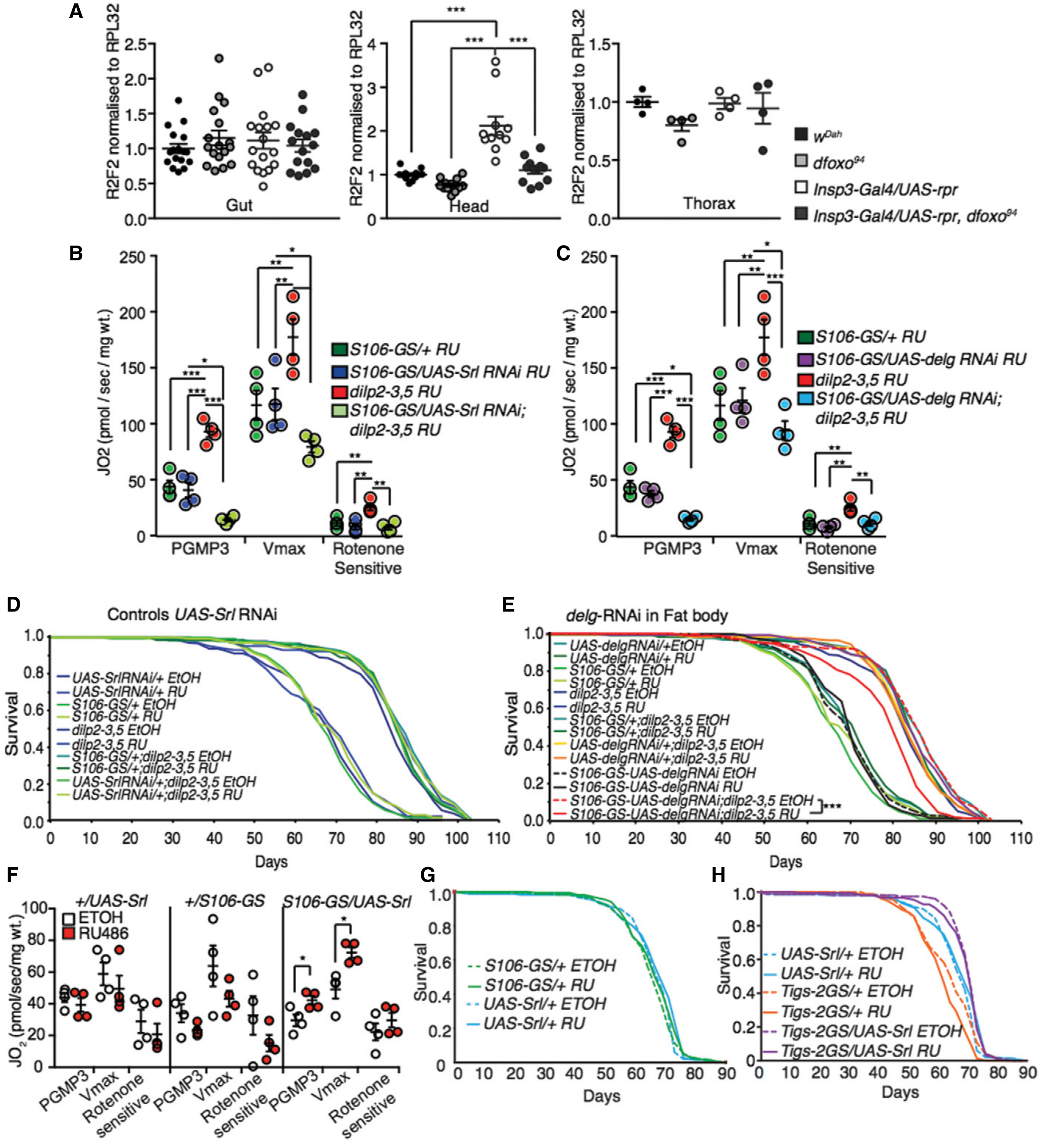

Figure EV4.

 © 2017 The Authors

Figure EV4. Reduced IIS does not regulate mtDNA levels in the gut or head.

A Relative mtDNA levels compared to nuclear DNA in the gut (*n* = 17), head (*n* = 12) and thorax (*n* = 4). Fat body mtDNA levels shown in Fig 5E, significance determined by two-way ANOVA.

B, C Fat body oxygen consumption normalized to dry wt. of flies with fat body-specific reduction of either (B) *Spargel* levels (*S106GS/UAS-Srl RNAi* + RU) (*n* = 4) or (C) *delg* (*S106GS/UAS-delg RNAi* + RU) in the presence and absence of *dilp2-3,5* and genetic controls (*n* = 4), significance determined by two-way ANOVA. Oxygen consumption was assessed by using substrates entering the level of complex I (PGMP3), complex I+II once uncoupled by CCP (Vmax) and rotenone-sensitive complex I+II + rotenone (*n* = 4), significance determined by two-way ANOVA.

D Lifespan analysis of genetic control flies with fat body-specific reduction in *Spargel* levels (*S106-GS/UAS-Srl RNAi* + RU) (Fig 4F) (*n* > 150).

E Lifespan analysis of *dilp2-3,5* mutant and control flies with reduced expression of *Delg* in the fat body (*S106-GS/UAS-Delg RNAi*) induced by RU or none induced (*n* = 150). Statistical significance was determined by Log Rank test.

F Fat body oxygen consumption normalized to dry wt. of flies with induced (RU) or non-induced (EtOH) fat body-specific over-expression of *Spargel* (*S106GS/UAS-Srl*) and genetic controls (*+UAS-Srl* and *+/S106GS*) (*n* = 4), significance determined by one-way ANOVA.

G Lifespan analysis of genetic control flies from over-expression of *Spargel*, specifically in the adult fat body (Fig 4G) (*n* = 150).

H Lifespan analysis of flies with induced (RU) or non-induced (EtOH) over-expression of *Spargel* in the adult gut (*Tigs-2GS/UAS-Srl*), along with genetic controls (*n* = 150).

Data information: Error bars indicate mean SEM (*$P < 0.05$; **$P < 0.01$; ***$P < 0.001$).

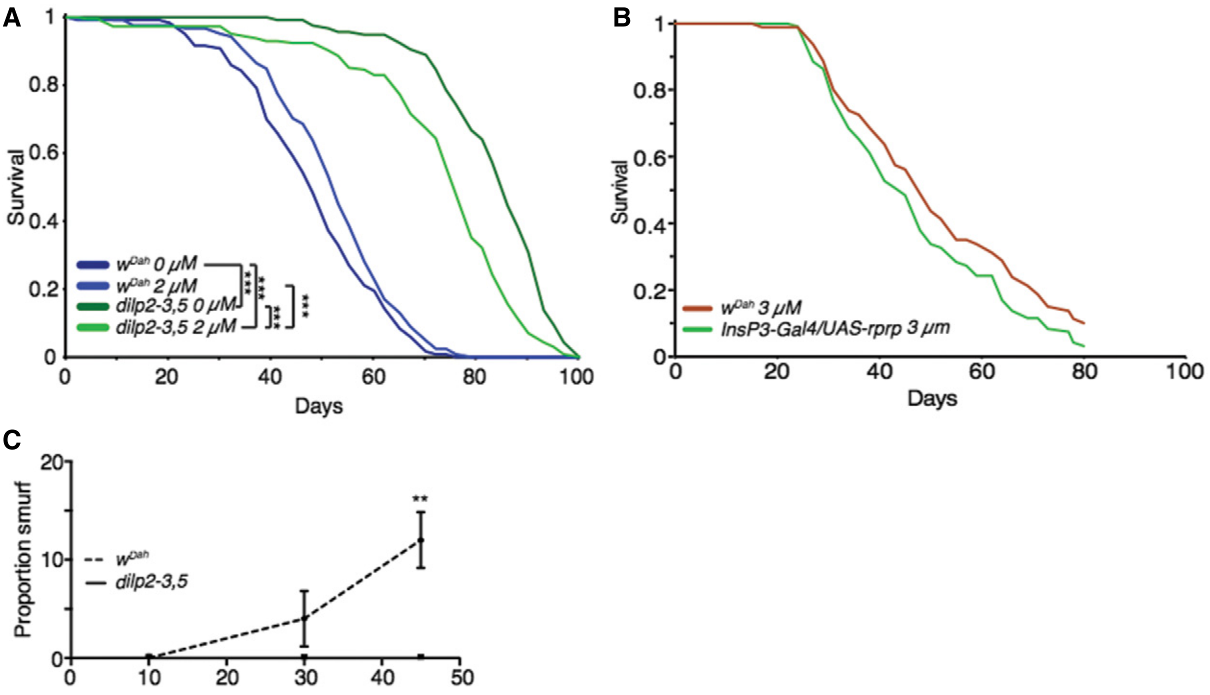

Figure EV5. Proteasome activity is necessary for IIS-mediated longevity, in an independent model of reduced IIS.

A Lifespan analysis of control flies (w^{Dah}) and *dilp2-3,5* mutant flies treated with 2 μM proteasome inhibitor (bortezomib) or vehicle (EtOH) (*n* > 125). Statistical significance was determined by Log Rank test.

B Lifespan analysis of wild-type flies (w^{Dah}) and mNSC-ablated flies treated with 3 μM proteasome inhibitor (bortezomib) (*n* > 80).

C Assessment of age-related gut integrity through "Smurf" assay at different ages (10, 30, 45 days) in wild-type flies (w^{Dah}) and *dilp2-3,5* mutant flies (*n* = 100), significance determined by *t*-test at each age.

Data information: Bars indicate mean SEM (**$P < 0.01$; ***$P < 0.001$). For associated lifespan, see Fig 6.

© 2017 The Authors

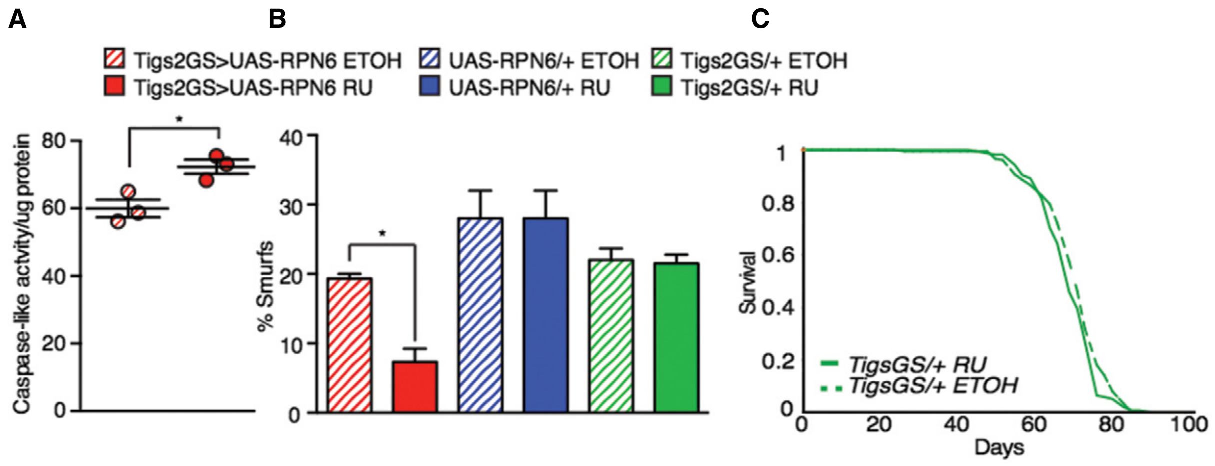

Figure EV6. Control lifespan analysis for adult gut-specific expression of proteasomal subunit RPN6, proteasomal activity and gut integrity.

A Gut proteasome activity (caspase-like) of *Tigs2-Geneswitch/UAS-RPN6* flies compared to *+/UAS-RPN6* control flies (n = 3), significance determined by t-test.

B Assessment of gut integrity though "Smurf" assay (65 days) between induced (RU treated) and uninduced (ETOH treated) *Tigs2-Geneswitch/UAS-RPN6* (n = 200) flies and induced (RU treated) and uninduced (ETOH treated) driver controls *Tigs2-Geneswitch/+* (n = 200) and *UAS* (*+UAS-RPN6*, n = 75) control flies.

C Gut-specific driver (*Tigs-Gal4-GS*/+) control lifespan in the presence of RU486 (200 µm) or vehicle (EtOH) ($n > 180$).

Data information: Bars indicate mean SEM (*$P < 0.05$).

 © 2017 The Authors

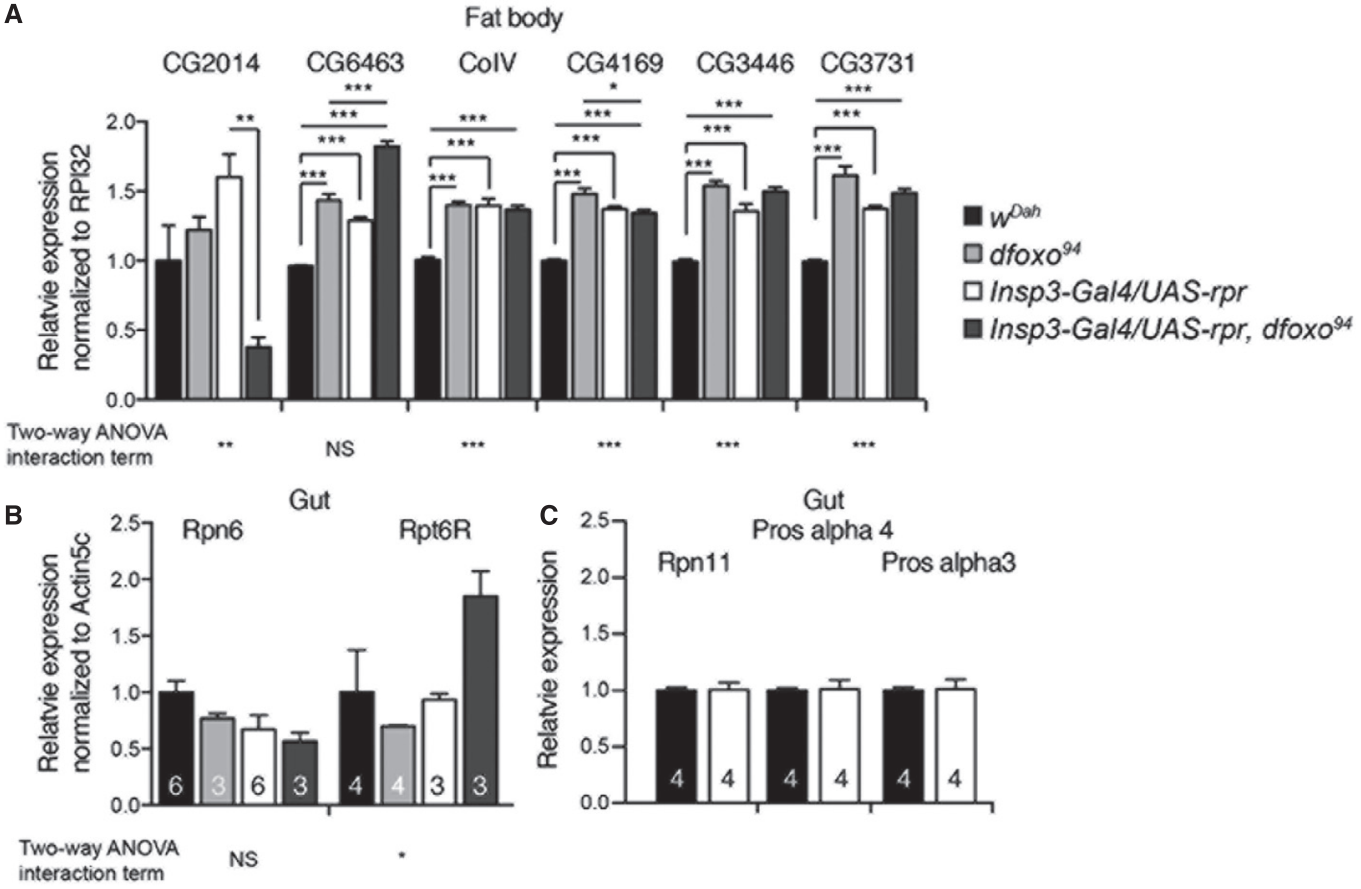

Figure EV7. qRT–PCR analysis of tissue-specific candidate gene expression in response to reduced IIS.

A–C qRT–PCR analysis shows gene expression levels of *CG2014, CG6463, ColV, CG4169, CG3731* in the fat body (A), or of *Rpn6, Rpt6R, Rpn11* and *Pros-alpha3* in the gut (B, C) of control (ω^{Dah}), *dfoxo* mutant (*dfoxo*^{94}), mNSC-ablated (*InsP3-Gal4/UAS-rpr*) and mNSC-ablated flies in the absence of *dfoxo* (*InsP3-Gal4/UAS-rpr, dfoxo*^{94}). Relative expression levels are normalized to either RPL32 (A) or Actin5c (B, C). Significance established by two-way ANOVA and *post hoc* pairwise tests (n = 3, or otherwise shown). Bars indicate mean SEM (*P < 0.05; **P < 0.01; ***P < 0.001).

© 2017 The Authors
